# Phase Angle as an Easy Diagnostic Tool of Meta-Inflammation for the Nutritionist

**DOI:** 10.3390/nu13051446

**Published:** 2021-04-24

**Authors:** Luigi Barrea, Giovanna Muscogiuri, Gabriella Pugliese, Daniela Laudisio, Giulia de Alteriis, Chiara Graziadio, Annamaria Colao, Silvia Savastano

**Affiliations:** 1 Centro Direzionale, Dipartimento di Scienze Umanistiche, Università Telematica Pegaso, Via Porzio, Isola F2, 80143 Napoli, Italy; 2 Centro Italiano per la Cura e il Benessere del Paziente con Obesità (C.I.B.O), Endocrinology Unit, Department of Clinical Medicine and Surgery, University Medical School of Naples, Via Sergio Pansini 5, 80131 Naples, Italy; giovanna.muscogiuri@gmail.com (G.M.); robiniapugliese@gmail.com (G.P.); dani.laudisio@libero.it (D.L.); chiaragraziadio@live.it (C.G.); operafederico2@gmail.com (A.C.); sisavast@unina.it (S.S.); 3 Unit of Endocrinology, Dipartimento di Medicina Clinica e Chirurgia, Federico II University Medical School of Naples, Via Sergio Pansini 5, 80131 Naples, Italy; dealteriisgiulia@gmail.com; 4 Cattedra Unesco “Educazione alla Salute e allo Sviluppo sostenibile”, University Federico II, 80131 Naples, Italy

**Keywords:** phase angle, meta-inflammation, Mediterranean diet, C-reactive protein, bioimpedance analysis, nutritionist

## Abstract

Phase angle (PhA), a noninvasive bioimpedance marker, is a useful tool for nutritional screening in several diseases. C-reactive protein (CRP), a strong risk factor for metabolic and cardiovascular diseases, is a commonly used biomarker of meta-inflammation. As both PhA and CRP are influenced by age, BMI, and nutritional status, and exhibit a clear sex dimorphism, we examined the association between PhA and CRP levels in 1855 subjects (680 males and 1175 females), aged 18–59 years, with BMIs ranging from 19.5 to 69.4 kg/m^2^, stratified according to sex. PhA values and CRP levels were significantly lower in females than males (*p* < 0.001), while the adherence to the Mediterranean diet (MD) was lower in males compared to females (*p* < 0.001). After adjusting for age, physical activity, BMI, waist circumference, and adherence to the MD, PhA remained negatively associated with CRP levels in both genders (*p* < 0.001). In the ROC analysis, PhA ≤ 5.5° in males and ≤5.4° in females were the threshold values predicting increased hs-CRP levels. These results suggested that PhA might represent a valid predictor of CRP levels in both sexes regardless of body weight and adherence to the MD, which avoids the collection of blood sampling and expensive biochemical assays.

## 1. Introduction

Several diseases are characterized by an underlying condition of low-grade, chronic inflammation, also known as meta-inflammation, which is associated with increased levels of inflammatory mediators, such as acute-phase proteins synthesized by the liver cells under the influence of cytokines [1,2,3].

C-reactive protein (CRP) is the acute-phase protein most studied as an inflammatory biomarker in relation to a variety of pathologic processes [4]. Elevated CRP levels are associated with oxidative stress and may contribute to cellular damage and cell death through apoptosis or necrosis [5]. However, monitoring CRP levels requires the collection of venous blood samples and access to a well-equipped laboratory, which can limit its evaluation in clinical practice. In addition, large epidemiological studies reported that there is a clear sex dimorphism in CRP levels [6,7,8].

Bioimpedance analysis (BIA) is a simple and validated approach to indirectly identify inflammatory biomarkers of cell death and cellular damage by evaluating individual fluid distribution and whole-body cell membrane quality [9]. In particular, the phase angle (PhA) is a raw parameter obtained from BIA and calculated from the arctangent of two direct parameters measured by BIA instruments that are the reactance (Xc) and resistance (R) ratio. PhA reflects cell membrane integrity and size, and it is used as a marker of body cell mass [10,11]. PhA also represents the water distribution between the extracellular water and intracellular compartments [12,13]. A smaller PhA suggests cell death, decreased cell integrity, and breakdown in the selective permeability of the cell membrane. Contrarily, a larger PhA suggests a great number of intact cell membranes, body cell mass, and healthy cell membranes [14,15]. In healthy individuals, gender, age, body weight, and nutritional status are the major predictors of PhA [11,16]. In this context, we previously reported that the Mediterranean diet (MD), a healthy anti-inflammatory dietary pattern also known to actively modulate cell membrane properties, was positively associated with PhA regardless of gender, age, and body mass index (BMI) [17]. Taken together with the aforementioned evidence, it is expected that altered measurements of the PhA may also be associated with inflammatory markers [11,18]. Previous clinical studies reported that PhA might represent a screening tool for the identification of inflammation in patients with obesity [19], polycystic ovary syndrome [20], hypovitaminosis D [21], hidradenitis suppurativa [22], psoriasis [23], cancer [24,25], and type 2 diabetes [26]. Furthermore, PhA also appears to be a useful biomarker for cachexia, sarcopenia, and malnutrition in hospitalized individuals with cardiovascular disease [27]. In addition, PhA is an important independent predictor of mortality [28,29].

The associations between PhA and oxidative stress and inflammatory biomarkers, including CRP, have been previously investigated in a group of older females, among which there was a negative association between PhA and CRP levels regardless of common modifiable and non-modifiable risk factors, such as age and body composition [30]. Very recently, we reported that PhA was inversely associated with CRP levels in adults affected by Prader–Willi syndrome, the most common genetic cause of obesity [31].

Although PhA and CRP levels are closely linked to the same pathophysiological processes, it is still unknown whether the association between PhA and CRP levels might be dependent on sex and adherence to the MD. Starting from this evidence, the goal of the present study was to fill this gap by examining the association between the PhA values and CRP levels as biomarkers of meta-inflammation in a large sex-stratified adult population sample.

## 2. Materials and Methods

### 2.1. Design and Setting

In this monocentric study, participants were enlisted from December 2011 to February 2021 at the Obesity Unit (C.I.B.O. and EASO center) at the Unit of Endocrinology, Clinical Medicine and Surgery Department, University “Federico II” of Naples. Some of the participants were also recruited during the OPERA prevention project [32]. This research was approved by Ethical Committee Federico II (n. 239/11) and was conducted following the Code of Ethics of the World Medical Association (Declaration of Helsinki) for human studies. All subjects were informed about the study design and purpose and gave informed consent.

### 2.2. Population Study

A total of 1855 adult participants, 680 males and 1175 females, aged 18–59 years, with BMIs ranging from 19.5 to 69.4 kg/m^2^, and only Caucasian individuals, were enrolled in this study. All individuals were healthy as ascertained by a participant questionnaire (absence of a clinical condition that potentially influences fluid balance, i.e., myocardial, endocrine, or renal diseases). All female participants were non-lactating and non-pregnant. Females of childbearing age were evaluated only in the follicular phase of the menstrual cycle. Full medical information, such as a drug use, was collected from all subjects enrolled in this study. The exclusion criteria included:

- Patients with the following diseases: type 1 and type 2 diabetes, cancer, anxiety or depression, diagnosed anemia, or any other metabolic diseases requiring a special nutritional approach. We also excluded individuals with hepatic or renal insufficiency, chronic inflammatory diseases, alcohol abuse, or on pharmacological therapy that could influence body weight;
- Occasional or current use of any of the medications that could influence fluid balance, or carbohydrate or lipid metabolism, e.g., anticonvulsants and psychotropic agents, non-steroidal anti-inflammatory drugs, laxatives, metformin, statins, and fish oil;
- Patients with endocrine disorders; in particular, females who had used oral contraceptives or reproductive hormone therapy in the past 3 months;
- Subjects with a history of intolerance or allergy to food belonging to the MD (including nuts, fish, and others);
- Individuals that for any reason followed a specific dietary regimen (hypocaloric diet, ketogenic diet, vegetarian diet, and others);
- Supplementation with any vitamins/minerals or antioxidants;
- Subjects who were simultaneously participating in other studies;
- Due to the potential possibility of interference with the device activity because of the field of current induced by the impedance measurements, we excluded patients with implanted pacemakers or defibrillators;
- Subjects who had suffered skin damage in the area where the BIA electrodes were attached;
- Underweight patients (BMI < 18.5 kg/m^2^).

### 2.3. Physical Activity and Smoking Habits

Through the administration of a standard questionnaire, as already reported in other studies [33,34], physical activity levels and cigarette smoking habits were assessed in all participants. Participants were classified into inactive or active subjects, based on the minutes of daily physical activity performed. In particular, those who practiced at least 30 min of daily aerobic physical activity were classified as active.

Similarly, the participants were divided into current smokers (when smoking at least one cigarette per day) or non-current smokers, those who do not smoke any cigarettes a day, as we reported earlier [35,36].

### 2.4. Anthropometric Measurements

Anthropometric measurements and assessment were done in all participants between 8 a.m. and 10 a.m., after an overnight fast. For weight and height measurement, a calibrated balance beam scale was used to the nearest 50 g (Seca 711; Seca, Hamburg, Germany) and a wall-mounted stadiometer to the nearest 1 cm. All participants wore light clothing and were without shoes when measuring. As previously reported [37,38,39] and in accordance with the WHO’s criteria [40], BMI was calculated by weight (kg) and height squared (m^2^). Participants were classified into five BMI classes: normal weight, overweight, grade I obesity, grade II obesity, and grade III obesity (BMI: 18.5–24.9 kg/m^2^, 25.0–29.9 kg/m^2^, 30.0–34.9 kg/m^2^, 35.0–39.9 kg/m^2^, and ≥40.0 kg/m^2^, respectively).

Waist circumference (WC), according to the NCHS, was obtained using a non-stretchable measuring tape to the closest 0.1 cm at the narrowest point. In patients with grade III obesity, or in subjects where no narrowest point of the waist was visible, WC was taken using a non-stretchable measuring tape to the nearest 0.1 cm at umbilical level [41].

### 2.5. Inflammatory Marker

In the morning (8.00–10.00 a.m.), and after an overnight fast of at least 8 h, the blood samples were collected and subsequently stored at −80 °C until processed.

Serum high-sensitivity (hs) CRP levels were analyzed by CardioPhase^®^ (Siemens Healthcare Diagnostics, Marburg, Germany), based on particle-enhanced immunonephelometry. The coefficient of variation (CV) of intra- and interassay was <7%.

### 2.6. Nutritional Parameters

As we reported earlier [42,43,44], the adherence to the MD was assessed by the PREvention with MEDiterranean Diet (PREDIMED) questionnaire, a previously validated 14-item questionnaire [45]. The PREDIMED questionnaire was performed by a certified clinical nutrition specialist with 5 years of experience through a face-to-face interview with all the enrolled individuals. Scores of one (Yes) and zero (No), were assigned for each of the 14 items of the PREDIMED questionnaire. The PREDIMED score was calculated from the sum of the scores of the 14 questions of the PREDIMED questionnaire. In accordance with the PREDIMED score, we divided the participants into three categories of adherence to the MD as follows: highest adherence, average adherence, and lowest adherence to the MD (PREDIMED score ≥ 10, 6–9, and 0–5, respectively) [45].

### 2.7. Body Composition and PhA

As previously reported [46,47,48,49], and according to the ESPEN [13], the same certified clinical nutrition specialist with 5 years of experience in the assessment of body composition performed body composition measurements using a BIA phase-sensitive system (BIA 101, RJL Akern Bioresearch, Florence, Italy, 800 µA current at a single frequency of 50 kHz) [50]. The BIA exam was performed by the same device to avoid interdevice and interobserver variability. Before applying the electrodes, subjects were asked to remove their shoes and socks (BIATRODES Akern Srl; Florence—Italy). According to Kushner [51], the contact areas of electrodes on the hand and the ipsilateral foot, were scrubbed with alcohol immediately before their placement.

PhA (°, degrees) was obtained by the formula: Xc/R*(180/π). Xc and R data were obtained under strictly standardized conditions: all individuals were supine with limbs slightly spread apart from the body and had refrained from exercising for six hours and eating and drinking within 24 h before testing.

### 2.8. Statistical Analysis

Data were analyzed using the SPSS Software and MedCalc^®^ package. The data distribution was evaluated by a Kolmogorov–Smirnov test. The abnormal data (age, weight, height, BMI, WC, hs-CRP levels, PREDIMED score, R, Xc, and PhA) were normalized by a logarithm. The differences in continuous variables between males and females were compared using the Student’s unpaired *t*-test (age, weight, height, BMI, WC, hs-CRP levels, PREDIMED score, R, Xc, and PhA), while the differences in multiple groups (BMI classes and PREDIMED categories) were analyzed by an ANOVA test followed by the Bonferroni post hoc test. The chi-square (χ^2^) test was used to determine the statistically significant differences in the frequency distribution, including sex, smoking, physical activity, BMI classes, and PREDIMED categories comparing males with females. The correlations between PhA with age, weight, height, BMI, WC, hs-CRP levels, PREDIMED score, R, Xc, and PhA were performed using Pearson *r* correlation coefficients. A partial correlation was performed to control for effects of confounding factors on PhA including age, sex, physical activity, BMI, WC, and PREDIMED score. A proportional odds ratio (OR) model, *p*-value, 95% confidence interval (CI), and R^2^ were used to assess the association between PhA with categorical variables included in this study (sex, smoking, physical activity, BMI classes, and PREDIMED categories). A multinomial logistic regression analysis, χ^2^, *p*-value, Akaike information criterion (AIC), and R^2^ were used to model to compare the relative predictive power of age, sex, physical activity, BMI, WC, hs-CRP level, and PREDIMED score associated with the PhA values. Finally, two receiver operator characteristic (ROC) curve analyses were performed to determine the area under the curve (AUC), criterion, sensitivity and specificity, standard error, and 95% CI as well as cut-off values for PhA (°) predictive of hs-CRP levels above the median value (2.33 ng/mL and 1.90 ng/mL, in males and females, respectively).

## 3. Results

The study population consisted of 1855 individuals, 680 males (36.7%) and 1175 females (63.3%), aged 18–59 years, with BMIs ranging from 19.5 to 69.4 kg/m^2^. There were 1191 physically active subject (64.2%), while there were 484 current smokers (26.1%). Regarding BMI classes, the most common was grade III obesity (518 subjects, 27.9%). In addition, based on PREDIMED categories, the majority of participants had a low–average adherence to the MD (39.0% and 38.7%, respectively), while only 22.3% of the study population reported a high adherence to the MD.

The descriptive characteristics, including age, lifestyle, anthropometric measurements, inflammatory marker, and nutritional and body composition parameters of the study population according to sex, are given in Table 1. In particular, there were no obvious sex differences in age and physical activity levels (*p* = 0.511 and *p* = 0.071, respectively). In addition, more of the female participants were non-smoking (*p* < 0.001) and presented grade III obesity (*p* < 0.001), despite a higher percentage of adherence to the MD (*p* < 0.001) compared to males. Females showed also lower hs-CRP levels (*p* = 0.003), lower Xc and PhA values, and higher R than males (Xc and PhA: *p* < 0.001, and R: *p* = 0.022).

PhA, according to smoking, physical activity, BMI classes, and PREDIMED categories, is showed in Table 2. PhA was significantly higher among physically active individuals (*p* < 0.001), in overweight subjects (*p* < 0.001), and in subjects with a high adherence to the MD (*p* < 0.001). No statistically significant difference was evident between smokers and non-smokers (*p* = 0.106).

### Correlation Analysis

The correlations between PhA and age, anthropometric measurements, inflammatory marker, and nutritional and body composition parameters are summarized in Table 3. All parameters evaluated in this study were significantly associated with PhA.

**Figure 1:**
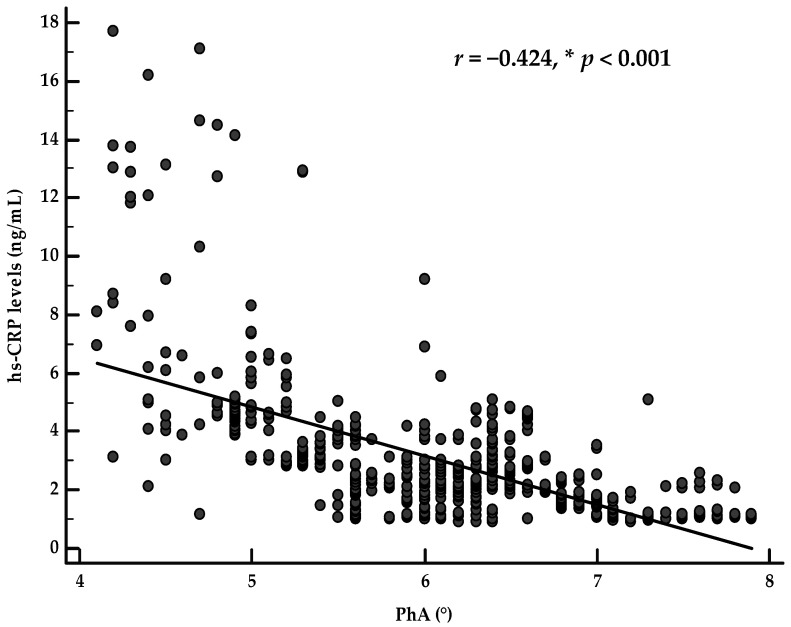
Correlation between PhA and inflammatory marker after adjusting for age, physical activity, BMI, WC, and PREDIMED score, in male participants. * A significant difference (*p* < 0.05).

**Figure 2:**
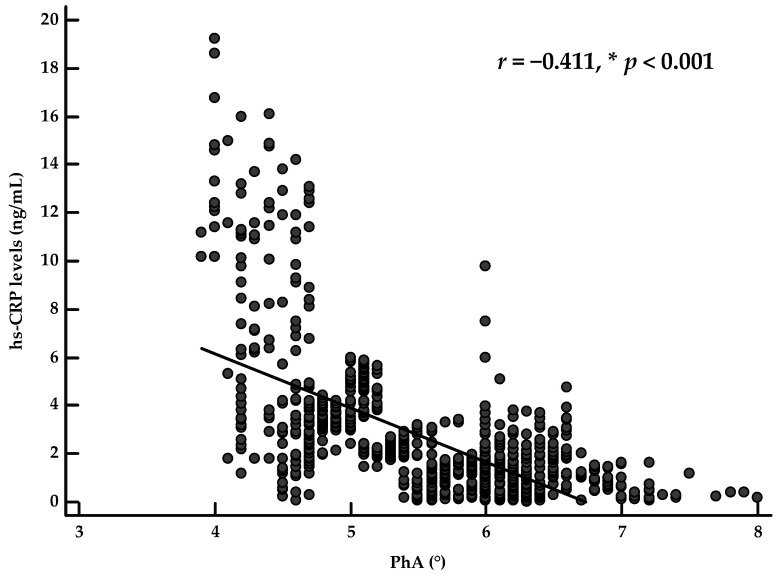
Correlation between PhA and inflammatory marker after adjusting for age, physical activity, BMI, WC, and PREDIMED score, in female participants. A * denotes a significant difference (*p* < 0.05).

When dividing the population according to sex, PhA was inversely associated with hs-CRP levels (*r* = −0.609, *p* < 0.001 and *r* = −0.614, *p* < 0.001 in males and females, respectively). Of interest, after adjusting for age, physical activity, BMI, WC, and PREDIMED score, the correlation between PhA and hs-CRP levels was still evident in males (*r* = −0.424, *p* < 0.001) and females (*r* = −0.411, *p* < 0.001), as shown in Figure 1 and Figure 2, respectively.

Table 4 reports the results of a bivariate proportional OR model performed to assess the association between PhA and categorical variables included in this study. PhA was significantly associated with all categorical variables (*p* < 0.001), except smoking (OR 0.90, *p* = 0.106). In particular, the highest PhA values were associated with males (OR 0.90, *p* = 0.106), physically active subjects (OR 1.63, *p* < 0.001), the overweight classes (OR 6.66, *p* < 0.001), and the highest adherence to the MD (OR 3.26, *p* < 0.001) (Table 4).

To compare the relative predictive power of age, sex, physical activity, BMI, WC, hs-CRP level, and PREDIMED score associated with the PhA values, we performed a multiple linear regression analysis using a model that included these variables. Using this model, PREDIMED score was entered at the first step, followed by sex, hs-CRP levels, BMI, and age (*p* < 0.001 for all), while physical activity and WC were excluded. The results are reported in Table 5.

**Figure 3:**
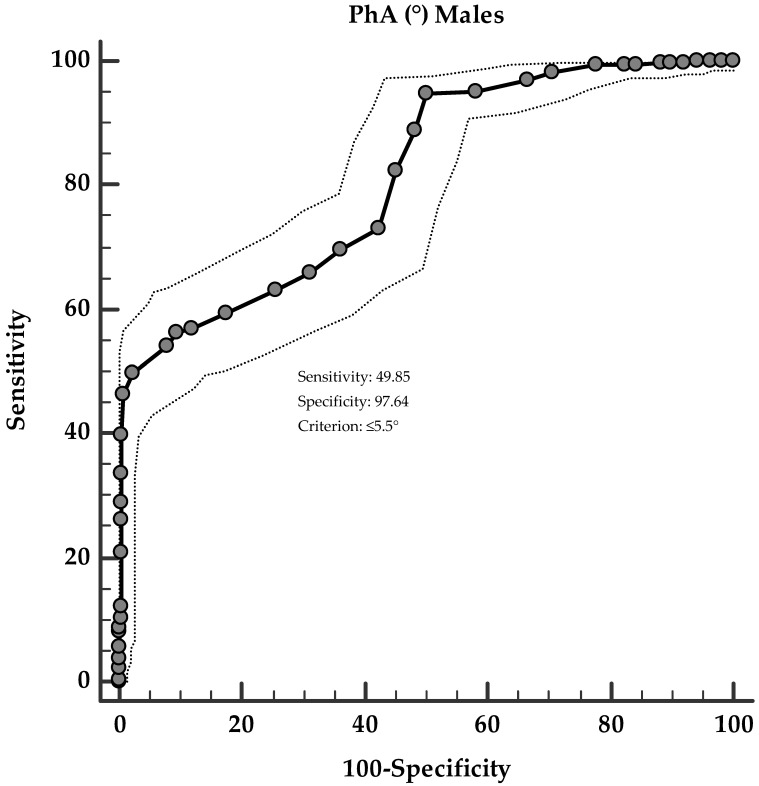
ROC for predictive values of PhA (°) in detecting the highest hs-CRP levels in males.

**Figure 4:**
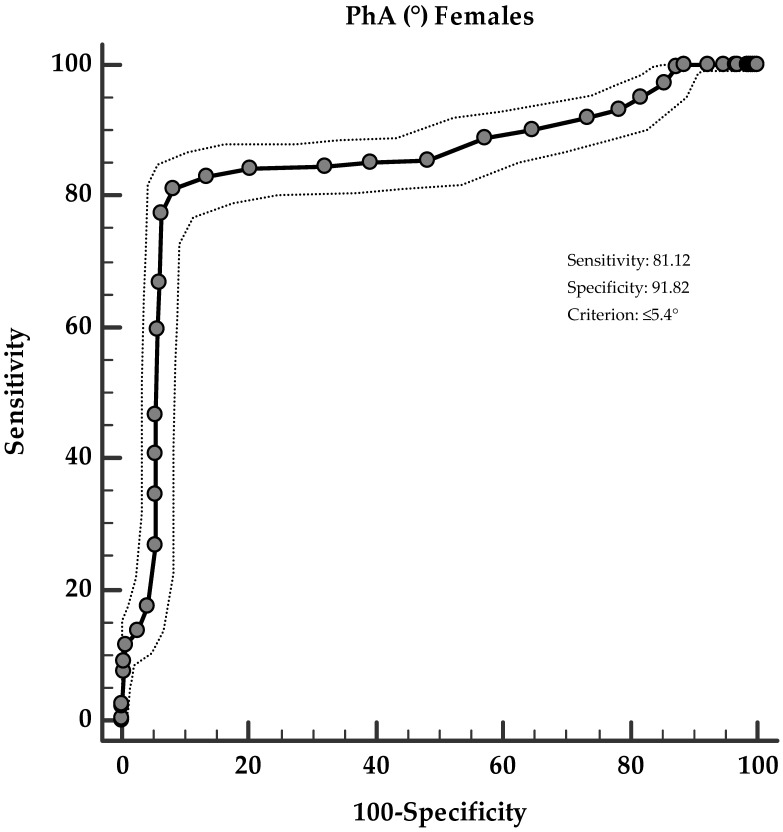
ROC for predictive values of PhA (°) in detecting the highest hs-CRP levels in females.

Two ROC analyses were then performed to determine the cut-off values of PhA according to the sex prediction of hs-CRP levels. In particular, in males, PhA ≤ 5.5° (*p* < 0.001, AUC 0.811, standard error 0.016, 95% CI 0.779 to 0.842; Figure 3), and in females, PhA ≤ 5.4° (*p* < 0.001, AUC 0.850, standard error 0.013, 95% CI 0.825 to 0.874; Figure 4), could serve as thresholds for significantly increased hs-CRP levels below the median values (2.33 ng/mL and 1.90 ng/mL, in males and females, respectively).

## 4. Discussion

The aim of the present study was to evaluate whether there was a sex difference in the association between PhA values and CRP levels in a large sample of the adult population. Although PhA was lower among females than males, male participants exhibited an unhealthy lifestyle, characterized by higher prevalence of smoking habits, higher WC measurements, and lower adherence to the MD, which was associated with higher CRP levels compared to the female counterparts. However, in both sexes, smaller PhA was associated with higher CRP levels, and this inverse association was also independent of other common confounding variables, such as age, physical activity, anthropometric measurements, and adherence to the MD. These findings indicated that in our adult population sample, PhA might represent a more reliable predictor of meta-inflammation than other common anthropometric measurements, such as BMI and WC. Based on the ROC curve analysis, we found that ≤5.5° in males and ≤5.4° in females were the cut-offs for the PhA to predict the highest CRP levels. To the best of our knowledge, to date, this is the first study assessing the association of PhA with a validated marker of inflammation, such as CRP, in a large sample of the adult population according to sex.

While the validity of CRP as a biomarker of meta-inflammation is well established, to date, the biological significance of PhA in meta-inflammation has yet to be clarified. Of interest, both PhA and CRP are influenced by age, BMI, and nutritional status, and exhibited a clear sex dimorphism. However, the influence of sex on the association between these variables, which are both linked to the meta-inflammation, has not yet been investigated.

PhA is a raw parameter derived from two direct values of the BIA, R and Xc, and it has been extensively used as an indicator of inflammatory status and cell membrane function, as well as a marker of nutritional status and dietary intake in different population studies [9,11,52,53]. In a healthy population, gender, age, and body weight are the major predictors of PhA [11,16]. Due to lower fat mass and higher muscle mass percentage, PhA is known to be higher in healthy males in comparison to females. Very recently, PhA has been proposed as a useful marker of inflammation and altered hydration status in patients affected with COVID-19 [54]. In detail, Cornejo-Pareja et al. reported that low PhA was a significant predictor of mortality risk in COVID-19, independent of age, sex, BMI, and comorbidities, suggesting that PhA assessment should be included in the routine clinical assessment of COVID-19 patients at increased mortality risk [54].

In addition, large epidemiological studies reported that there is a clear sex dimorphism in CRP levels [6,7,8]. Changes in PhA and CRP also depend on age, as PhA decreases and CRP levels increase with advancing age [15]. In fact, it has been consistently reported that the aging process is accompanied by meta-inflammation, characterized by a high level of circulating inflammatory biomarkers, particularly CRP, which promotes cellular damage and cardiovascular risk [55,56]. On the other hand, BMI variably affected the functional status of cell membranes, for both fat and muscle mass. For these reasons, there is a biphasic association of PhA with age and BMI. In particular, there are an inverse correlation with older age and obesity classes and a positive correlation with younger and normal weight/overweight subjects [57,58]. In line with other authors [57,59,60,61,62], in our population sample, PhA and CRP levels had a significant and opposite association with age.

As an adjunctive finding, among the confounding variables possibly affecting the association between PhA and CRP, we also included adherence to the MD, a healthy, anti-inflammatory dietary pattern known to actively modulate cell membrane properties [17,63]. The ESPEN strongly recommended PhA as a prognostic nutritional measure [64,65], and reliable marker for the identification of inflammation in subjects at risk of impaired nutritional status [11,66,67]. In this context, we have previously shown that in both sexes, a higher adherence to the MD was associated with larger PhA, uncovering a new benefit of the MD for PhA as a marker of inflammation and cell membrane integrity [17]. In the present study, we further provided evidence on the positive association between PhA and adherence to the MD, which was also the major determinant of the PhA values.

Several pieces of evidence support a role for PhA as a screening tool for the identification of inflammation in patients with a number of disease conditions, including obesity [19], polycystic ovary syndrome [20], hypovitaminosis D [21], hidradenitis suppurativa [22], psoriasis [23], cancer [24,25], and type 2 diabetes [26]. The inverse association shown between PhA and CRP levels can be explained by the fact that although the inflammatory processes are the first line of defense against invading pathogenic microorganisms and a crucial process for the repair of tissue injury, an exaggerated or unregulated prolonged inflammatory process can induce tissue damage [5,68]. In particular, it is well established that CRP, as an acute marker of inflammation, is deposited at sites of inflammation and tissue damage [5,69]. Our results are in agreement with previous evidence that showed the association of PhA with inflammatory biomarkers in several diseases [18,30,70,71]. In particular, Stobäus et al., in 777 (aged > 18 years) hospitalized patients in a retrospective analysis, reported that CRP levels exhibited a significant influence on PhA and concluded that next to the established predictors, such as gender, age, and body weight, inflammation in sick individuals also strongly affected PhA values [18]. Subsequently, Tomeleri et al. examined the association of PhA with inflammatory biomarkers in 155 older women, with BMIs varying from normal weight to grade I obesity [30]. The authors reported that, after controlling for potential covariates, such as age and number of diseases, PhA exhibited a negative association with all inflammatory biomarkers, particularly CRP [30]. The results of these two studies corroborate the findings of our two previous studies [23,31]. In the first study, conducted in 180 adult patients with psoriasis, an inflammatory skin disease that is associated with increased CRP levels, we reported the first evidence that psoriatic patients presented lower PhAs compared to controls, with a novel negative association between PhA and CRP levels independent of BMI [23]. More recently, we also confirmed the negative association between PhA and CRP after adjustment for sex, BMI, and WC in patients with Prader–Willi syndrome, which is associated with a high prevalence of metabolic dysfunction and meta-inflammation [31]. Given the obsessive–compulsive characteristics of this syndrome, which are commonly associated with poor compliance with blood sampling, the usefulness of PhA as a non-invasive marker of inflammation was strongly suggested in these patients.

The findings of the present study lend further support to the association between PhA and CRP and suggested a novel use of PhA as an alternative marker of meta-inflammation. This finding could be of particular interest when considering that monitoring CRP involves invasive collection of blood samples and expensive biochemical assays, which may limit its use in clinical practice. In addition, this study extends the knowledge on the association between PhA and CRP by demonstrating that this association was independent of sex, as suggested by the substantial similarity of the cut-off values to predict the CRP levels in both male and female participants.

### Limitations and Strengths of This Study

Firstly, the cross-sectional design of the study does not allow us to identify causality in the relationship between PhA and CRP, thus limiting the external validity in other populations. However, considering the independence of the common confounding variables included in this study, our data suggested that this inverse association might still be generalized, to some extent, to other populations. Second, we did not analyze other inflammatory markers. However, it is widely accepted that CRP represents the most studied inflammatory biomarker in relation to a variety of pathologic processes [4]. Therefore, we used an hs-CRP method, which allows an acceptable precision level of <0.3 mg/L in detecting gender differences in CRP median values. Third, the proposed sex cut-off point of PhA for identifying the highest CRP levels should be validated by further clinical trials. A major point of strength of this study is the actual sample size of our population, which also shared the same geographical area and the same food availability. This allowed us to improve the homogeneity of the study sample. Another major strong point is the adjustment with a number of covariates commonly affecting PhA and CRP, including sex and adherence to the MD. In addition, to minimize the interoperator variability, only one clinical nutritionist performed and interpreted the BIA measurements, and the PhA values were obtained using the same BIA instrument. In fact, it should be mentioned that differences in PhA values obtained from BIA single frequency vs. BIA multifrequency instruments from different manufacturers have been observed [29]. Finally, considering that, according to the indications reported in ESPEN guidelines, the use of BIA in clinical practice is not indicated for routine assessment in patients with an unbalanced state of hydration and underweight subjects (BMI < 19 kg/m^2^) or those with grade III obesity (BMI > 40 kg/m^2^) [13], in the present study, we chose to evaluate the PhA as the most clinically relevant and direct impedance measure of BIA, which is not influenced by altered hydration in the algorithmic computations to assess free fat mass, body cellular mass, and other body composition compartments, or by height and weight [72,73].

## 5. Conclusions

This was the first report showing that in a large population of adult subjects of both sexes, the negative association between PhA and CRP levels was independent of covariates commonly affecting PhA and CRP, including sex and adherence to the MD. As possible translational applications, these findings further support that PhA may be an easy and reliable measure to detect meta-inflammation, which could avoid blood sampling and expensive biochemical assays.

## Figures and Tables

**Table 1:** Differences in study parameters between males and females.

Parameters	Males Subjects *n* 680 (36.7%)	Females Subjects *n* 1175 (63.3%)	* *p*-Value
Age (Years)	34.8 ± 11.3	34.4 ± 11.2	0.511
Lifestyle characteristics			
Smoking (Yes)	253 (37.2%)	231 (19.7%)	χ^2^ = 67.86, *p* < 0.001
Physical activity (Yes)	225 (33.1%)	439 (37.4%)	χ^2^ = 3.23, *p* = 0.071
Anthropometric parameters			
Weight (kg)	104.8 ± 25.9	95.3 ± 24.7	<0.001
Height (m)	1.76 ± 0.05	1.63 ± 0.07	<0.001
BMI (kg/m^2^)	33.8 ± 8.1	35.6 ± 8.6	<0.001
Normal weight (*n*, %)	107 (15.7%)	174 (14.8%)	χ^2^ = 0.22, *p* = 0.638
Overweight (*n*, %)	161 (23.7%)	161 (13.7%)	χ^2^ = 29.18, *p* < 0.001
Grade I obesity (*n*, %)	132 (19.4%)	197 (16.8%)	χ^2^ = 1.88, *p* = 0.169
Grade II obesity (*n*, %)	133 (19.6%)	272 (23.1%)	χ^2^ = 3.05, *p* = 0.081
Grade III obesity (*n*, %)	147 (21.6%)	371 (31.6%)	χ^2^ = 20.73, *p* < 0.001
WC (cm)	111.1 ± 24.4	101.2 ± 24.0	<0.001
Inflammatory marker			
hs-CRP levels (ng/mL)	2.9 ± 2.3	2.5 ± 2.7	0.003
Nutritional parameters			
PREDIMED score	6.6 ± 3.1	7.3 ± 3.0	<0.001
Low adherence to the MD	318 (46.8%)	406 (34.6%)	χ^2^ = 26.48, *p* < 0.001
Average adherence to the MD	264 (38.8%)	453 (38.6%)	χ^2^ = 0.01, *p* = 0.947
High adherence to the MD	98 (14.4%)	316 (26.9%)	χ^2^ = 37.99, *p* < 0.001
Body composition parameters			
R (Ω)	471.5 ± 90.2	481.2 ± 85.7	0.022
Xc (Ω)	49.9 ± 10.3	46.5 ± 9.4	<0.001
PhA (°)	6.1 ± 0.8	5.6 ± 0.7	<0.001

* A significant difference (*p* < 0.05).

**Table 2:** PhA in the study population according to smoking, physical activity, and BMI classes, and PREDIMED categories.

Parameters		PhA (°)	*p*-Value
Smoking	Yes (*n* 484, 26.1%)	5.7 ± 0.83	0.106
	No (*n* 1371, 73.9%)	5.8 ± 0.81	
Physical activity	Yes (*n* 664, 35.8%)	6.0 ± 0.77	<0.001
	No (*n* 1191, 64.2%)	5.6 ± 0.82	
BMI classes	Normal weight (*n* 281, 15.1%)	6.1 ± 0.52	<0.001
	Overweight (*n* 322, 17.4%)	6.6 ± 0.59	
	Grade I obesity (*n* 329, 17.7%)	6.0 ± 0.68	
	Grade II obesity (*n* 405, 21.8%)	5.5 ± 0.75	
	Grade III obesity (*n* 518, 27.9%)	5.2 ± 0.61	
PREDIMED categories	Low adherence to the MD (*n* 724, 39.0%)	5.2 ± 0.71	<0.001
	Average adherence to the MD (*n* 717, 38.7%)	6.0 ± 0.73	
	High adherence to the MD (*n* 414, 22.3%)	6.3 ± 0.55	

**Table 3:** Correlations between PhA and age, anthropometric parameters, inflammatory marker, nutritional and BIA parameters.

PhA (°)		
Parameters	*n* 1855	
	*r*	*p*-Value
Age (Years)	−0.114	<0.001
Anthropometric parameters		
Weight (kg)	−0.469	<0.001
Height (m)	0.190	<0.001
BMI (kg/m^2^)	−0.579	<0.001
WC (cm)	−0.421	<0.001
Inflammatory marker		
hs-CRP levels (ng/mL)	−0.550	<0.001
Nutritional parameters		
PREDIMED score	0.593	<0.001
Body composition parameters		
R (Ω)	−0.211	<0.001
Xc (Ω)	0.501	<0.001
PhA (°)	−0.550	<0.001

**Table 4:** Bivariate proportional odds ratio model to assess the association between PhA and categorical variables included in this study.

Parameters	PhA (°)			
	OR	*p*-Value	95% IC	R^2^
Sex (Male)	0.41	<0.001	0.36–0.47	0.10
Smoking (Yes)	0.90	0.106	0.79–1.02	0.01
Physical activity (Yes)	1.63	<0.001	1.44–1.84	0.04
BMI classes				
Normal weight (*n*, %)	1.85	<0.001	1.57–2.17	0.03
Overweight (*n*, %)	6.66	<0.001	5.32–8.35	0.20
Grade I obesity (*n*, %)	1.49	<0.001	1.29–1.74	0.02
Grade II obesity (*n*, %)	0.55	<0.001	0.47–0.63	0.04
Grade III obesity (*n*, %)	0.21	<0.001	0.18–0.25	0.20
PREDIMED categories				
Low adherence to the MD	0.18	<0.001	0.15–0.21	0.27
Average adherence to the MD	1.76	<0.001	1.56–1.99	0.05
High adherence to the MD	3.26	<0.001	2.77–3.84	0.13

**Table 5:** Regression analysis model with the PhA as a dependent variable to estimate the predictive value of age, sex, physical activity, BMI, WC, hs-CRP level, and PREDIMED score.

Parameters	Regression Analysis			
	R^2^	β	t	*p* Value
PREDIMED score	0.352	0.59	31.7	<0.001
Sex	0.504	−0.39	−23.9	<0.001
hs-CRP levels (ng/mL)	0.598	−0.35	−20.8	<0.001
BMI (kg/m^2^)	0.613	−0.17	−8.7	<0.001
Age (Years)	0.625	−0.11	−7.4	<0.001
Variables excluded: physical activity and WC				

## Data Availability

Results attained in this study are included in the manuscript. Individual data are not publicly available due to ethical restrictions.
